# Maintenance of gut microbiome stability for optimum intestinal health in pigs – a review

**DOI:** 10.1186/s40104-022-00790-4

**Published:** 2022-12-07

**Authors:** Santi Devi Upadhaya, In Ho Kim

**Affiliations:** Department of Animal Resource and Science, Dankook University, No.29 Anseodong, Cheonan, 31116 Choongnam South Korea

**Keywords:** Microbial diversity, Nutritional intervention, Perturbation, Resilience, Swine gut microbiome stability

## Abstract

Pigs are exposed to various challenges such as weaning, environmental stressors, unhealthy diet, diseases and infections during their lifetime which adversely affects the gut microbiome. The inability of the pig microbiome to return to the pre-challenge baseline may lead to dysbiosis resulting in the outbreak of diseases. Therefore, the maintenance of gut microbiome diversity, robustness and stability has been influential for optimum intestinal health after perturbations. Nowadays human and animal researches have focused on more holistic approaches to obtain a robust gut microbiota that provides protection against pathogens and improves the digestive physiology and the immune system. In this review, we present an overview of the swine gut microbiota, factors affecting the gut microbiome and the importance of microbial stability in promoting optimal intestinal health. Additionally, we discussed the current understanding of nutritional interventions using fibers and pre/probiotics supplementation as non-antibiotic alternatives to maintain microbiota resilience to replace diminished species.

## Introduction

The swine gut microbiome is a complex and dynamic ecosystem harboring immensely diverse microbiota including bacteria, viruses, archaea, and fungi that ideally reside symbiotically in the gut of host animals. Among the microorganisms, the number of bacteria outnumbers other microorganisms [1, 2]. There is a co-existence of several hundred anaerobic bacterial species in the caecum and colon of swine [3, 4] ranging between 10^11^ and 10^12^ CFU/g digesta [5], whereas in the stomach and small intestine, a relatively lower numbers of bacteria (10^7^–10^9^ CFU/g fresh matter) are found [6]. These bacteria which inhabit the gastro intestinal tract (GIT) from birth significantly impact animal health, since they protect against the pathogens forming a key barrier, provide essential nutrients to the host from fermentation processes [7, 8] and play a pivotal role in maintaining the host physiological homeostasis, in promoting immune system development, and in regulating host metabolism [9–11]. Although the bacterial component of the microbiome is the major component affecting gut microbiome, the ability of the fungal microbiome, mycobiome has also been found to alter gut microbial community structure and cause disease [12–15]. Commensal fungi and viruses may also cause the alteration in the severity of disease and modification of immunological responses [16–19].

Gut microbiota composition varies among individuals and throughout the growth state, and is dependent on host genotype and environmental factors. Early microbial exposure, diet, age, breed, and antibiotics have all been implicated to the onset and maintenance of microbial diversity in the human gut [20]. The disruption of the gut ecosystem by perturbations would cause significant decreases in functional richness and microbial diversity as well impairment in metabolic functions. The loss of diversity due to the altered composition of microbes is called “dysbiosis” and can impact the immune system resulting in the emergence and outbreak of diseases and growth deprivation in pigs [21, 22]. The fundamental paradigm shift in our understanding of microorganisms in the GIT has become evident. For instance, all eukaryotes are meta-organisms and it is now accepted that they must be considered together as an inseparable functional unit [23]. This concept also takes into consideration that a tiny fraction of microorganisms consists of pathogens in the microbiome.

The microbiome robustness, the maintenance of diverse and functional microbiota in GIT is crucial for effective swine production. The microbiome robustness depends on the diversity of the microbiome, so it is not enough just to have the presence of a few different beneficial microbes. Accordingly, new strategies are required to manipulate the gut microbiome to prevent or revert unhealthy states caused by perturbations. This paper highlights the importance of microbial stability and nutritional intervention to maintain the stability of microbiota that inhabit within the gut microbiome. Dietary manipulation through the alterations of diet composition [24, 25], nutritional concentrations [26], energy resources [27], and diet types [28] had been widely proven to shape the gut microbiota communities. For conducting this review, literature search was done using the web of science database and data collection was based on more than 140 peer reviewed articles. The search words for this review article were ‘dietary intervention, microbial diversity, perturbation, resilience, stability, and swine gut microbiome’.

## Overview of swine gut microbiome

The intestinal microbiota in swine contributes in maintaining its good health of host and producing meat for human consumption [29–31]. Among all the microbes, bacteria accounts for the major share of microbiota (> 98% of the entire microbiota) in pig’s microbiome [32] and are influential in the digestion and metabolism of nutrients, immune homeostasis as well as energy supply [33–35].

The diverse microbial community originated from the sow and/or the environment rapidly colonizes the sterile GIT of newly born piglets [5, 36]. The initial microbial exposure primarily occurs at birth via urogenital and environmental exposure and at ingestion of colostrum and milk throughout lactation [37]. Immediately after birth, the GIT of piglets are mainly colonized by facultative aerobes or anaerobes. For instance, Swords et al. [38] reported that the sterile colon at birth was initially dominated by facultative anaerobes that stabilized at 10^9^–10^10^ bacteria/g colonic contents within 12 h from birth subsequently followed by the domination of obligate anaerobes within 48 h after birth making up more than 90% of the microflora thereafter. Some other studies showed that GIT of piglets within 6 h after birth was colonized by bacteria belonging to Streptococcaceae family which became most numerous during 1 to 3 days of birth and were gradually replaced by Lactobacillaceae and Clostridiaceae because of secondary colonization [39]. The dominant bacterial genera found during the suckling period are reported to be *Clostridium*, *Bacteroides* and *Bifidobacterium* [38].

Before weaning, the microbiota remains quite stable in piglet GIT after the initial colonization [40, 41]. Very early and sudden weaning stage of piglets aggravates the qualitative and quantitative alterations of gut microbiota, which may increase pathogenic microorganisms [42]. Besides the diet change from milk to solid food, separation from the dam and co-mingling with other littermates induce a lot of stress to the piglets and it provokes changes in the gut microbiota [42] as well as deprivation in feed intake and growth [43, 44]. More specifically, weaning-associated starvation results in shifts in microbial communities in the GIT that become unstable and less diverse due to the reduction in fermentable substrates [45]. The weaning transition is characterized by a loss of microbial diversity, a decrease in the abundance of bacteria belonging to the *Lactobacillus* group and an increase in the abundance of facultative anaerobes, including bacteria belonging to the Enterobacteriaceae, Proteobacteriaceae, Clostridiaceae and Prevotellaceae families [46, 47]. Moreover, the phylogenetic composition of fecal microbial community was dominated by Bacteroidetes, Firmicutes, Proteobacteria, and Spirochaetes at the phylum level and, at the genus level, *Prevotella*, *Lactobacillus*, and *Treponema* were the three most abundant genera [48, 49].

The meta-analysis conducted by Holman et al. [50] using 20 publicly available data sets from high-throughput 16S rRNA gene sequence studies revealed that the core genera *Prevotella*, *Clostridium*, *Alloprevotella*, *Ruminococcus*, and the RC9 were detected in 99% of the faecal samples obtained from commercial swine worldwide. The symbiosis of these core microbiome plays an important role in regulating nutrient metabolism and immunity of the host, ultimately contributing to the health and production of pigs [51, 52]. In a recent study, Li et al. [53] identified a “core” microbiome of 69 bacterial features that were present in all the physiological stages of pigs (lactation, nursery, growing, and finishing). In agreement with the findings of Holman et al. [50] most of these bacterial features were associated with the order Clostridiales, Bacteroidiales, and Lactobacillales and the top three families were Prevotellaceae, Ruminococcaceae, and Lactobacillaceae. Although *Megasphaera* and *Prevotella* spp. were present during all the growth stages, others such as Clostridiaceae and Bacteroidetes were not noticeable at lactation and nursery stages but emerged rapidly and became the dominant taxa at the growing and finishing stages [53].

### Impact of various factors on the gut microbiome in swine

The distribution and composition of gut microbiota may be influenced by various factors as follows:

#### Birth weight

The gut microbiota and metabolic status in the piglets are affected by their birth weights indicating that suckling period might be critical for modulating the gut microbiota in low-birth-weight piglets [54].

#### Physiological stage

Among several factors, physiological stage is considered as one of the determinant factors affecting the colonization and stabilization of gut microbiota in neonatal piglets [55] and the abundance of bacterial diversity is also influenced by age [56]. During the weaning at 21–28 days of age, the change in diet, as well as other environmental factors induce several stressors leading to significant alterations in the composition of swine gut microbiota and the pathways associated with nutrient metabolism [57].

#### Sex

The other determinant factor is sex. For instance, a higher abundance of Veillonellaceae, *Roseburia*, *Bulleidia* and *Escherichia* was seen in boars whereas the relative abundance of *Treponema* and *Bacteroides* was observed in gilts suggesting the influence of sex hormone, specifically androgen, in gut microbial composition [58]. A negative correlation was found between *Treponema* and androgen metabolites which is consistent with the reports that demonstrated the inhibition of *Treponema* growth due to higher level of testosterone [59]. However, the gut microbial structure of castrated boars was of higher similarity to gilts indicating this shift in microbiota composition of the boars towards that of gilts might be linked with inadequate secretion of androgen hormone due to castration in boars [58].

#### Breeds

A distinct gut microbiota composition is found in different breeds of pigs [60]. For instance, Landrace displayed a higher abundance of cellulolytic bacteria, indicating this breed has a better ability in fiber digestion [61] and Yorkshire showed reduced Firmicutes and greater Bacteroidetes concentrations, whereas Tibetian pigs had greater concentrations of bacteria from Elusimicrobia, Fibrobacteres and Spirochaetes [62] suggesting that microbiome composition may be affected depending on where the breed is originated or raised. The reduced Firmicutes/Bacteroidetes ratio in the Yorkshire pigs exhibited apparent differences compared to Rongchang and Tibetain pigs [62]. The various compositions of the intestinal microbiome can influence the usage of the host energy and nutrients [63]. Therefore, difference of microbial community could contribute to the quality and quantity of production in different breeds.

#### Different intestinal segments/contents

The intestinal tract of pigs is segmented into different compartments based on the differences in anatomical structures, physiological functions, and microbiota communities. The small intestine which is again divided into duodenum, ileum and jejunum mainly host microorganisms that are involved mainly in the digestion and absorption of the proteins, lipids, amino acids, monosaccharides, and some oligosaccharides. On the contrary, the large intestine (colon and cecum) is the habitat for microorganisms which play the role of the degradation of nutrients such as insoluble cellulose that are not digestible in the small intestine [64]. Furthermore, the number of microorganisms is higher in large intestine compared with the small intestine [5], and there is significant variation in microbial composition of the ileum as compared to that of the cecum and colon. For instance, in the ileum, the genera *Escherichia-Shigella* (23.1%), *Terrisporobacter* (17.9%), *Romboutsia* (13.7%) and *Clostridium sensustricto* (12.9%) are more abundant than in the cecum and colon. In cecum the three most prevalent genera are *Alloprevotella* (7.2%), *Lactobacillus* (5.0%), and the Prevotellaceae NK3B31 group (4.4%) whereas in the colon, the 3 most prevalent genera are *Streptococcus* (10.4%), *Lactobacillus* (8.8%), and *Clostridium* (8.0%) [65]. The intestinal microbiota can vary in animals based on biogeographic and geographic distributions [66, 67]. The geographical differences significantly affected the distribution of the phyla Actinobacteria, Verrucomicrobia, Firmicutes, and Fibrobacteres [68]. However, despite the same biogeographical area, the composition and abundance of gut microorganisms attached to the digesta or intestinal mucosa were different [69].

## Stability and diversity of gut microbiome

**Fig. 1:**
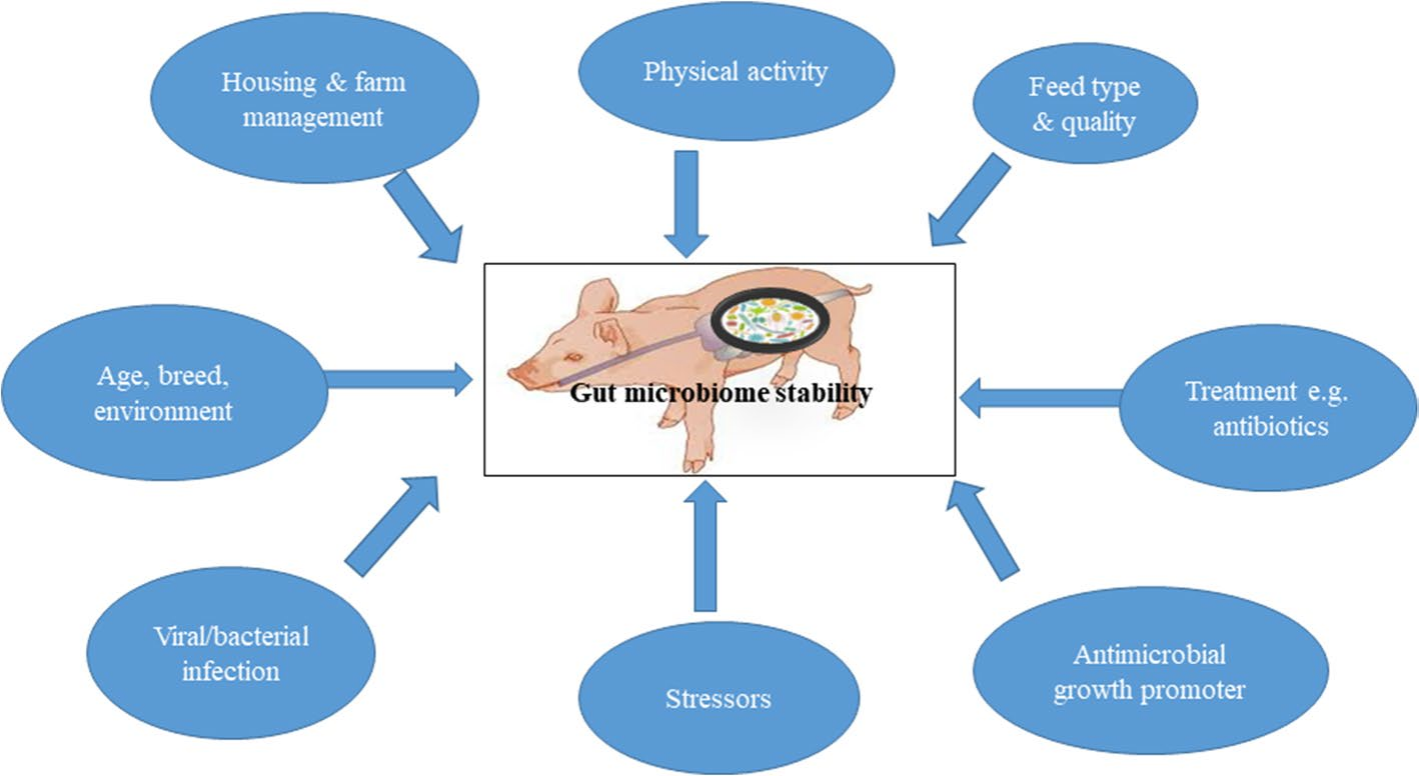
The increase in the risk of losing gut microbiome stability is influenced by several factors including therapeutic and sub-therapeutic antibiotics administration, feed types, physical activity, farm management, barn environment, age, breed, microbial infection and stressors

Stability is one of the essential ecological characteristics of the gut microbiome. The gut microbiome shows dynamic equilibrium and remains in its stable ecological state unless it is perturbed [70]. Nevertheless, the relative abundance of each microbe fluctuates over time and varies between and within individuals over the course of their lives [20, 71]. The microbial stability is influenced by several factors as shown in Fig. 1.

Generally, the gut microbiota is resilient when perturbed and allows the host to keep key species for a long period of time [72]. Thus, resilience is the property of an ecosystem to resist changes under stress or to quickly and fully recover from the perturbations [73]. However, the duration and severity of the perturbations can determine their impact on microbial community. The severe and intense external perturbations would alter the stable microbiota to unstable or transient state leading to an alternative stable state or unhealthy state associated with diseases. If the host acquires unhealthy microbiota having high self-regeneration or resilience potential, it may contribute to chronic microbial associated diseases [74]. Moreover, previous studies have suggested that very low diversity in a microbiome is a good predictor of poor health status [63, 75]. Thus, a healthy functional microbiome should comprise not a single static state but rather a dynamic ecosystem having the ability to recover to an equilibrium state after stress and perturbation [76].

**Fig. 2:**
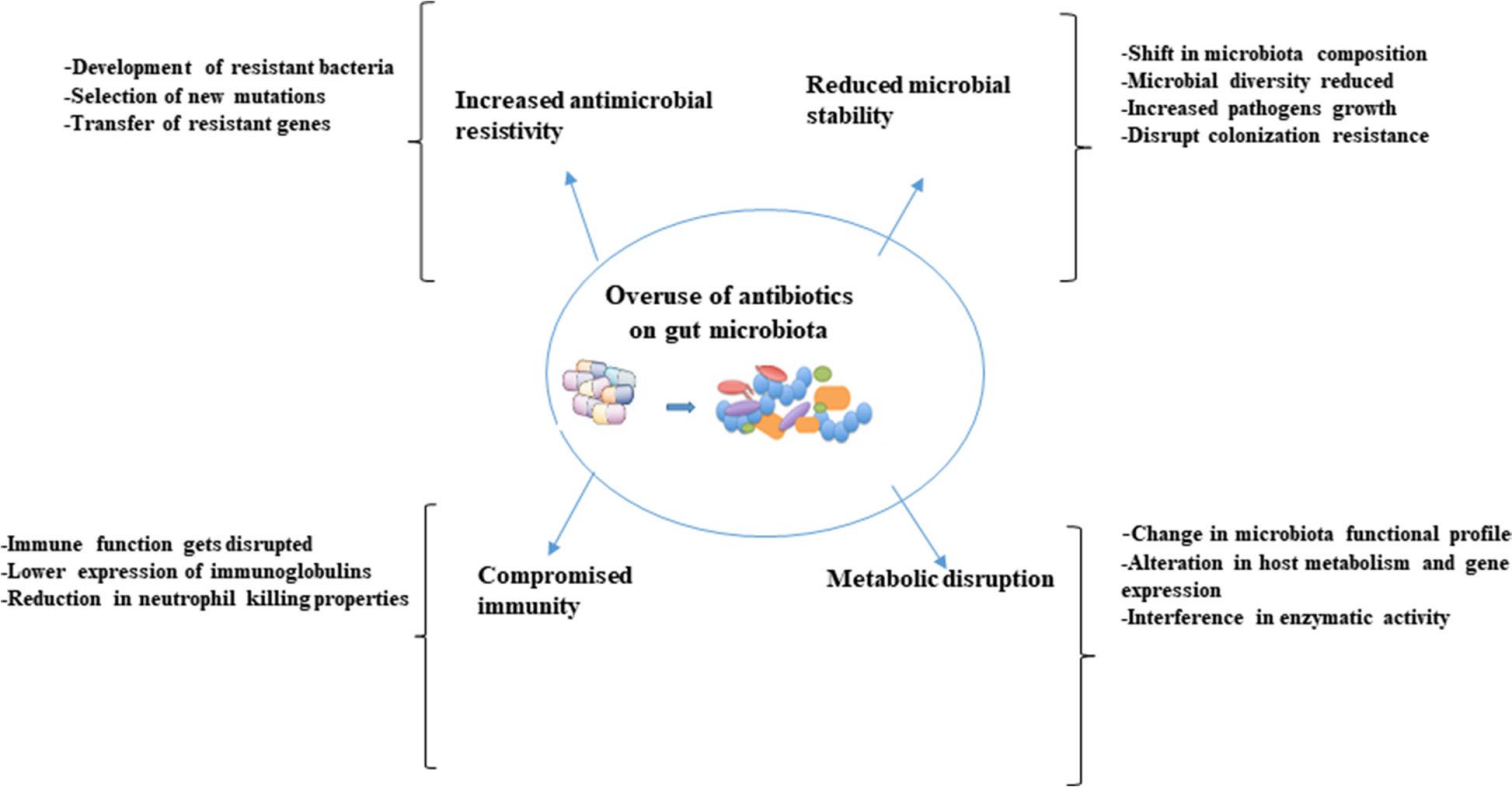
The overuse of antibiotics on swine gut microbiota has detrimental effects on the host health resulting in the loss of microbial stability and diversity, increased microbial resistance, compromised immunity, and metabolic disruption

The development of an unhealthy state of the gut is reported to be due to the drastic changes in dietary patterns, microbial infections and the extensive use of antibiotics [77] resulting in significant variation in compositional and/or functional microbiome, with marked decrease in diversity [78]. Diversity of the gut microbiota is likely very important to animal health [79]. The decrease in diversity consequently leads to the reduction of beneficial microorganisms and expansion of pathogenic microbes [80, 81]. The unhealthy states of the gut microbiome due to perturbation can either be temporary or it may develop into a permanent unhealthy state with negative implications. The most drastic perturbations to the gut microbiome are induced by prolonged antibiotic therapy that affects not only the targeted pathogens but also other members of the microbiota [82]. Furthermore, in the last few decades, antibiotics were not only used for therapeutic purpose but also as a growth promoter. The imprudent use of antibiotics as a therapeutic agent or a growth promoter over time has shifted the gut microbial population affecting its stability and diversity and has increased microbial resistance [83, 84] thereby affecting gut health consequently leading to adverse effects on the overall health of the animals as well as humans. For instance, administration of lincomycin (0.1%) through feed to the finishing pigs daily for 1–2 weeks resulted in the relative abundance of pathogenic microbes such as species of *Escherichia*-*Shigella*, *Clostridium*, and *Corynebacterium* but reduction of fiber degrading bacterial species such as *Treponema, Succinivibrio, Fibrobacter*, and *Cellulosilyticum* [85]. With the change in microbial community, lincomycin-administered swine microbiota showed deficiency in polysaccharide degradation and an increase in metabolic pathways related to peptidoglycan maturation and CMP-legionaminate biosynthesis and this pathway is linked with the adherence of pathogenic bacteria to mammalian cell surfaces [86] Thus, by impacting the composition of the microbial community, antibiotics alter microbiota functionality and the metabolites produced [87]. The detrimental impacts of prolonged use of antimicrobials on GI microbiota and host health are presented in Fig. 2.

In addition, the impact of antibiotic therapy is greatly influenced by ages of the studied populations, the chemical nature, pharmacokinetic and pharmacodynamics properties of the drug, target spectrum, route of administration and excretion, dose and duration, as well as the residing gut microbiota [88]. In a recent study, Gao et al. [89] demonstrated that in-feed administration of 200 mg/kg ampicillin, 5 mg/kg gentamicin, 40 mg/kg metronidazole modified GI microbial population structure and function in 42-day-old ileal-cannulated pigs. In addition, the reduction in *Lactobacillus* and *Bifidobacterium* abundance and increase in the abundance of *Shigella species* by 256-fold compared to the control pigs were also noted with the administration of these antibiotics [89]. Similarly, Li et al. [90] noted that the oral feeding of amoxicillin (30 mg/kg/d) twice daily to neonatal piglets during the first 14 days of age impacted developing gut microbiota and reduced the genes associated with short-chain fatty acid signaling and pancreatic development.

A mixture of olaquindox, kitasamycin, and oxytetracycline calcium (50 mg/kg each) administration as in-feed antibiotics to neonatal piglets has been reported to reduce the relative abundance of beneficial *Lactobacillus *species and increase the relative abundance of potentially pathogenic *Streptococcus suis* in both the small intestine and stomach lumen resulting in dysbiosis [91]. In growing piglets, antimicrobial administration induced alterations in microbiota composition in both abundant and less abundant GI microbiota. For instance, a higher relative abundance of *Lactobacillus*, *Eggerthella*, *Acetanaerobacterium*, and *Sporacetigenium* genera was observed in piglets receiving feed supplemented with tylosin (40 g/t feed) compared to control piglets [92].

The impacts of in-feed sub-therapeutic concentrations of two common antimicrobials such as tylosin (44–11 mg/kg feed) and chlortetracycline (5.5 mg/kg feed) during weaning, starter and growing-finishing periods on swine GIT microbiota composition have shown variable results. For example, tylosin administration resulted in a major shift in the relative abundance of several taxa, while chlortetracycline administration resulted only in minor alterations [93]. Similarly, administration of vancomycin and metronidazole in mice by oral gavage have different effects on *Clostridium difficile*, where only vancomycin had an obvious impact on microbial community structure [94].

To overcome the adverse effects of therapeutic and sub-therapeutic antibiotic administration in pigs gut microbiome, several possible alternatives have been mentioned [95–97]. In the following section, we will focus on the nutritional intervention (mainly dietary fiber), and feed additives, e.g., pre/probiotics supplementation to increase the microbiota diversity in the intestine of pigs thereby increasing resilience.

## Impact of nutritional intervention on gut microbiome

### Dietary fiber

Dietary fiber mainly constitutes non-starch polysaccharides (NSPs) such as (cellulose, arabinoxylans, chitins, pectins and beta-glucans), lignin and resistant starch [98]. These NSPs are naturally occurring compounds and are present in all plant-based feedstuffs including cereals, tubers, and agro-industrial byproducts [99]. The inclusion of dietary fiber in monogastric animal diets has gained considerable attention due to its potential beneficial effects on gut health and welfare, even though some adverse effects on nutrient and energy digestibility are exhibited [100]. Since pigs cannot degrade non-starch polysaccharides due to the lack of endogenous enzymes, the NSP and resistant starch escapes degradation in the small intestine and reach the lower part of the GIT being available for microbial fermentation [101]. Jha et al. [99], noted that fibers impact the composition and function of the microbiota, in monogastric animals especially the production of short-chain fatty acids. By increasing the proportion of defatted rice bran in the basal diet, the relative abundance of specific microbiota was found to be enhanced resulting in β-diversity variation in growing-finishing pigs [102]. The microbial process of fiber fermentation is variable due to the range of fiber sources and the physicochemical properties of that fiber. It has been reported that fibers fermented at a slow rate results in increased stool output, diluted colonic contents and production of distal colonic short chain fatty acid (SCFA), which is a major energy source for colonocytes [103–105]. A complex mix of dietary fibers providing a wide range of structures and monosaccharide units increase the microbiota diversity [106]. A recent study in Durco × Bamei crossbred pigs fed the basal diet supplemented with 10%, 17% and 24% dietary fiber (fermented silage broad bean) significantly altered the diversity of the bacterial community. The abundance of Bacteroidetes and *Turicibacter* increased with high dietary fiber in cecum and jejunum respectively resulting in alteration of concentration of their metabolites such as bile acids, fatty acids, carbohydrates and carbohydrate conjugate, and organic acids which may potentially influence nutrition absorption [107]. Tang et al. [108] suggested that adding fibers (Broad bean straw silage) to the basal diet significantly increased the α-diversity of the bacterial community in the jejunum and cecum, while the β-diversity decreased of Durco × Bamei crossbred pigs. Consequently, among the most abundant bacterial genera in the cecum, the relative abundance of unidentified *Prevotellaceae* and *Oscillibacter* increased with the increase in dietary fiber, while the richness of *Romboutsia, Intestinibacter*, and *Faecalibacterium*, decreased with the increase in dietary fiber. An earlier study had demonstrated that the gut microbiota of mice challenged with antibiotic returned to pre-challenge state by feeding fiber-enriched diet whereas the antibiotic challenged mice fed a low fiber diet lost their microbial diversity [109] suggesting that fibers have a direct effect on improving microbiota resilience.

### Feed additives (prebiotics and probiotics)

The use of feed additives such as pre/probiotics specifically at post-weaning, have been implemented to minimize the weaning-induced stress and improve microbiome status. Prebiotics are special non-digestible fibers that influence the composition and/or activity of the gastrointestinal microbiota and induce positive effects on host well-being and health [110, 111]. The prebiotics provides a substrate to be fermented by the gut beneficial microbiota. The inclusion of prebiotics in swine diets stimulates the proliferation and metabolic activity of beneficial microbes, contributing to a stable microbial ecosystem [112]. The most widely accepted prebiotics are lactulose, inulin, fructo-oligosaccharides (FOS) and galacto-oligosaccharides (GOS). Oligosaccharides which are short chain prebiotics have also been shown to be a potential alternative to in-feed antibiotics in young piglets due to their effect on the gut microbiota by providing a substrate for beneficial microorganisms [113]. Several studies in swine investigated the effect of prebiotics on the GIT microbiota in piglets around weaning period during previous years. For instance, Konstantinov and co-workers [114, 115] demonstrated that weaning piglets fed diet supplemented with a mix of sugar beet pulp, inulin, lactulose and wheat starch affected the composition of microbiota in the gut. Moreover, the fermentable carbohydrates having the ability to enhance colonic microbial stability and diversity simultaneously enhanced the growth of *Lactobacillus sobrius* [115]. *Lactobacillus sorbius* is found to colonize abundantly in the ileum of pigs where it exerts probiotic activity resulting in the prevention of epithelial damage by enteropathogenic *E. coli* as well as improvement in the daily weight gain of piglets [116–118]. In another study, Jiao et al. [119] demonstrated that increasing dose of cello-oligosaccharide supplementation resulted in increased *Lactobacillus* proportions and a reduction in potential pathogenic groups such as *Clostridium* in the weaning pigs suggesting the use of prebiotics as a promising approach to alleviate the post-weaning intestinal tract disorders.

Probiotics have been suggested and used as alternatives to antibiotic as a remedy to post-weaning diarrhea and as growth promoters [120–122]. Probiotics are direct-fed microbial which when administered in sufficient amounts confer health benefits to the host [123] and consist of organisms such as bacteria cells, yeast cells, or a blend of the two which modulate the gastrointestinal microbiota so as to improve the health of the host. The mechanism of action by probiotic has been suggested to be due to the suppression of pathogens, intestinal microbial communities’ manipulation, and immunomodulation, stimulation of epithelial cell proliferation and differentiation and fortification of the intestinal barrier [124]. *Lactobacillus* species, *Bacillus* species, *Bifidobacterium, Enterococcus faecium*, and *E. coli* have been developed as probiotics to promote the growth performance, mucosal immunity and epithelial function as well as to inhibit growth of pathogenic bacteria in swine [125–127]. The probiotic *L. sobrius* was found to be effective in the reduction of the *E. coli* F4 colonization and weight gain improvement of infected piglets [118]. The exopolysaccharide (EPS) secreted by lactic acid bacteria had exclusive properties in modifying the gut microbiota [128]. EPS have shown the potential to act as prebiotics to promote the increase of probiotics, providing support for the adhesion of probiotics in the GIT and their long-term survival, necessary for their effective propagation. It also acts as a source of carbon, helping the growth and colonization of gut bacteria by feeding them nutrients [129]. The supplementation of *L. rhamnosus* LB1 has been reported to alleviate ETEC’s adverse effects in pigs by improving host immune response, and restoring intestinal integrity [130]. However, in previous study, the dietary supplementation with *Lactobacillus rhamnosus* GG (originally used for human subjects) reduced the growth performance and impaired the health of *Escherichia coli* F4-challenged piglets [131]. Thus, the probiotic effects rely on the specific bacterial isolates suggesting the need of host target-specific probiotic strain [132]. Walsh et al. [133] reported that *Salmonella*-challenged pigs fed probiotics complex consisting of *Enterococcus faecium*, *Bacillus subtilis*, and *Bacillus licheniformis* in drinking water (10^9^ cfu/L for each strain of bacteria) showed no *Salmonella* in feces at 5 d post challenge. Lu et al. [134] indicated that probiotic complex supplementation including *Enterococcus faecium* DSM 7134, *Bacillus subtilis* plus *Lactobacillus paracasei* regulated the composition of the intestinal microbiota. Naqid et al. [135] demonstrated that *Lactobacillus plantarum* (B2984) strain supplementation into the feed of weaned piglets orally challenged with *S*. Typhimurium resulted in significant increase in immunoglobulins concentrations compared to their control counterparts. In our previous study, it was found that by supplementing the diet of *Salmonella*-challenged weaning pigs with *Bacillus*-based probiotics (*B. subtilis* RX7 and *B. methylotrophicus* C14 strains) boosted the immune system by improving RBC, lymphocyte, IgG, and IgM concentrations in the blood [136]. Beyond the restoration of the microbiota composition, due consideration must be given on how to minimize the effects of perturbed microbiota on the host. Dysbiosis often results in the emergence and outbreak of diseases [137] and increased gut permeability [138], consequently impacting the gut microbiota negatively. To overcome these adverse effects, interventions with probiotics complex with proven anti-inflammatory properties or having the ability to strengthen the gut barrier functions may be a good complementary strategy to improving the microbiota by acting on the host physiology [139–142]. Based on the reports from different studies, our recently published review work [120] summarized that the impact of pre/probiotics in reducing the stress associated with weaning is due to the antimicrobial effects of these feed additives against the harmful microbes and favoring the growth of beneficial microorganisms, enhancement of mucin expression thereby blocking *E. coli* invasion or due to the modulatory effect in the intestinal tight junction proteins thereby enhancing intestinal barrier functions as well as immune functions.

## Conclusion

This study highlights the importance of microbial stability and reviews the nutritional intervention to maintain the stability of microbiota in GIT. There is immense diversity in swine gut microbiota that varies between individuals and the gut microbiota can fluctuate over time, especially during early development, and under diseased conditions. The gut microbiota and their stability are influenced by host genetics, age, breed, physical activity, microbial infection, stressors, diet quality and types, antibiotics etc. Short-term perturbation resulting from dietary changes may restore microbiota to its original state, but long-term disturbances, such as antibiotic administration, could cause alterations in microbial diversity. Furthermore, the disturbance of the gut microbiota equilibrium through long-term perturbations, such as inflammation, poor feed or antibiotic, could lead to dysbiosis resulting in compromised immunity and consequently causing diseases. Thus, the landscape of stable states for the microbiota and its implications for resilience is an important research direction. To overcome the adverse effect of the perturbations especially due to long term antibiotic use, the nutritional intervention with feed additives could be one of the possible solutions among others. The selected feed additives including dietary fiber, prebiotics, and probiotics were focused in this review. The reported positive impact of these feed additives indicate that these feed additives can be effectively used in maintaining gut microbiome robustness and stability for optimum intestinal health in pigs although some inconsistent effects of probiotics are reported suggesting to select probiotics or probiotic complex that are host target-specific probiotic strain, safe and have proven anti-inflammatory and gut strengthening properties. Furthermore, due consideration must be given to the dose, efficacy as well as safety on the usage of these feed additives.

## Data Availability

Not applicable.

## Abbreviations

EPA: Exopolysaccharide
FOS: Fructo-oligosaccharides
GIT: Gastrointestinal tract
GOS: Galacto-oligosaccharides
IgG: Immunoglobulin G
IgM: Immunoglobulin M
NSP: Non-starch polysaccharides
RBC: Red blood cell
SCFA: Short chain fatty acid
